# Preferential coupling of an incident wave to reflection eigenchannels of disordered media

**DOI:** 10.1038/srep11393

**Published:** 2015-06-16

**Authors:** Wonjun Choi, Moonseok Kim, Donggyu Kim, Changhyeong Yoon, Christopher Fang-Yen, Q-Han Park, Wonshik Choi

**Affiliations:** 1 Department of Physics, Korea University, Seoul 136-701, Korea; 2 Department of Bioengineering, University of Pennsylvania, Philadelphia, Pennsylvania 19104, USA; 3 Center for Molecular Spectroscopy and Dynamics, Seoul 136-701, Korea

## Abstract

Light waves incident to a highly scattering medium are incapable of penetrating deep into the medium due to the multiple scattering process. This poses a fundamental limitation to optically imaging, sensing, and manipulating targets embedded in opaque scattering layers such as biological tissues. One strategy for mitigating the shallow wave penetration is to exploit eigenchannels with anomalously high transmittance existing in any scattering medium. However, finding such eigenchannels has been a challenging task due to the complexity of disordered media. Moreover, it is even more difficult to identify those eigenchannels from the practically relevant reflection geometry of measurements. In this Letter, we present an iterative wavefront control method that either minimizes or maximizes the total intensity of the reflected waves. We proved that this process led to the preferential coupling of incident wave to either low or high-reflection eigenchannels, and observed either enhanced or reduced wave transmission as a consequence. Since our approach is free from prior characterization measurements such as the recording of transmission matrix, and also able to keep up with sample perturbation, it is readily applicable to *in vivo* applications. Enhancing light penetration will help improving the working depth of optical sensing and treatment techniques.

Disordered media such as human skin, white powders and fogs, although they are not strong absorbers, appear to be opaque. This is due to a large number of scattering events occurring inside these media, which disperse the directionality of the wave propagation and attenuate wave transmission. Similar to the electric resistors in which scattering of free electrons with fixed atoms causes electrical resistance, light scattering in disordered media causes resistance to the energy flow^1^. Considering that the scattering in these media is a random process, it is not straightforward to control the wave transmission. However, recent studies have shown that the transmittance of wave energy can be controlled^2^,^3^,^4^,^5^ under conditions where coherence time of light source is much longer than the transit time broadening induced by a disordered medium, and the medium is stationary during the recording of the wave. Under these conditions, scattering process is not stochastic, but deterministic. As a result, one can shape the incident wave to alter the interference among multiple-scattered waves^6^,^7^,^8^.

The random matrix theory (RMT) has provided a theoretical framework for a systematic way of controlling wave interference^9^,^10^. The response of a disordered medium to an incident wave is linear as long as the intensity of the wave is low enough not to induce nonlinear optical processes. The disordered medium then acts as a linear transformation operator, which can be described by a transmission matrix *t* connecting orthogonal modes at the input plane to those of transmitted waves. According to RMT, one can identify the transmission eigenchannels of the medium by performing the singular value decomposition on the transmission matrix:


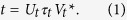


Here, *V*_*t*_ and *U*_*t*_ are unitary matrices whose columns correspond to transmission eigenchannels at the input and output planes, respectively, and * indicates conjugate transpose. The *τ*_*t*_ is a diagonal matrix and the physical meaning of its diagonal element is the amplitude transmittance of the corresponding eigenchannel. A remarkable point is that amplitude transmittance of each transmission eigenchannel has a broad distribution ranging from 0 close to unity. This indicates that the transmittance can vary significantly depending on the choice of the transmission eigenchannel. Since the transmittance of individual eigenchannel is determined by the degree of constructive wave interference at the output plane of the medium, wavefront shaping to transmission eigenchannels provides a way of controlling wave interference.

Recent experimental studies have verified the existence of transmission eigenchannels. In optics, Vellekop *et al.* first verified the existence of open eigenchannels, the eigenchannels with unity transmission, by analyzing the transmission enhancement of a point-optimization process^2^. Later, our group demonstrated that the coupling of waves into individual transmission eigenchannels significantly modify wave transmission. Popoff *et al.* reported the enhanced/reduced transmission by the feedback control of total transmitted intensity and explained its connection to the limited channel coverage^11^,^12^. In these studies, the eigenchannels were identified by the measurement of a transmission matrix of the medium^13^,^14^. In microwave regime, Shi *et al.* measured transmission matrix of both diffusive and localized media and explored the properties of maximum transmission eigenchannels^15^. The same group studied the contribution of modes to the total transmission^1^. Most of these previous studies concerned the transmission eigenchannels determined by the transmission matrix. However, the measurement of reflection is the only possible mode of detection in most real practice, which necessitates the investigation of the reflection eigenchannels. In the ultrasound regime, Aubry *et al.* measured reflection matrix and investigated the eigenvalue distribution depending on the arrival times^16^. It is rather in an early stage in optics, but Popoff *et al.* measured backscattering matrix for a weakly scattering medium and demonstrated selective wave focusing into a strongly scattering particle embedded in the medium^17^. And our group also reported the recording of the time-resolved reflection matrix for either a limited number of output modes^5^ or a large number of output modes^18^. While there has been an interesting study that demonstrated the interferometric control of absorption for a flat and clean absorbing layer^19^, there has been little study concerning the control of reflectance for a highly disordered medium.

In this Letter, we interrogated reflected waves from a disordered medium to get access to the reflection eigenchannels rather than transmission eigenchannels. Especially, we showed the possibility of finding an incident wave that preferentially couples into reflection eigenchannels of either high or low reflectance. We verified the purification of reflection eigenchannels from incident waves by the direct comparison with reflection eigenchannels obtained from reflection matrix. The preferential coupling to the reflection eigenchannels of low (high) reflectance has led to the increase (reduction) in transmission. We demonstrated transmission increase by more than twice the average transmittance. Since the proposed method makes use of reflected waves, it will potentially be useful for real practical applications of imaging and sensing targets embedded in scattering layers.

We describe the theoretical background of purifying reflection eigenchannels in a way to gradually refine incident waves. Similar to the transmission matrix, one can describe the way a scattering medium reflects incoming wave by a reflection matrix, *r*. This reflection matrix then connect reflected wave, ***E***^*r*^, and incident wave, ***E***^*in*^, by the simple relation, ***E***^*r*^ = *r**E***^*in*^. The *r* can be constructed from complex field maps of reflected waves for a set of orthogonal modes at the input plane. Then, the reflection eigenchannels of the medium can be found by performing the singular value decomposition on *r*:


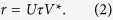


Here, *V* and *U* are unitary matrices whose columns correspond to reflection eigenchannels at the input and output planes, respectively. The *τ* is a diagonal matrix whose diagonal element indicates the amplitude reflectance of the corresponding eigenchannel. One can sort eigenchannels in the descending order of their amplitude reflectance and assign an eigenchannel index, *i*, to the amplitude reflectance of each eigenchannel, *τ*_*i*_, such that *τ*_*i*_ ≥ *τ*_*j*_ for *i* < *j*. For the *τ*_*I*_, the *i*^*th*^ column vectors of *V* and *U*, ***v***_*i*_ and ***u***_*i*_, are the corresponding eigenchannels at the input and output planes, respectively. The arbitrary incident wave is the linear superposition of the input eigenchannels and thus can be written as


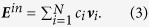


Here *c*_*i*_ is the coefficient determined by the scalar product between ***E***^*in*^ and ***v***_*i*_, and *N* is the total number of eigenchannels. Statistically, the incident wave is composed equally of all the eigenchannels such that the ensemble average of the absolute square of *c*_*i*_ is the same regardless of *i*. After the multiplication of *r* on this incident wave, one can obtain the following reflected wave.


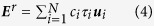


Note that the coefficient of each eigenchannel is weighted by the amplitude reflectance of the corresponding eigenchannel. In other words, high-reflection eigenchannels are weighted after one-time reflection.

Let us then consider the case in which the incident wave is decomposed into two parts, i.e. part *I* and part *II* as shown in the Fig. 1(a). Then the wave coming from each part is the linear superposition of eigenchannels as described in Eq. (3). When the phase of the part *II* is shifted by Δ*ϕ*, then the reflected wave of the combined wave is written as


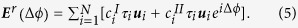


Since the eigenchannels are orthogonal with respect to one another, the interference occurs only between eigenchannels with the same index. Therefore, the total intensity of the reflected wave is given as
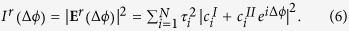
Since the intensity contributed by each eigenchannel is a sinusoidal function of Δ*ϕ* with the same period of 2π, the total intensity is also a sinusoidal function of Δ*ϕ*. Therefore, the total intensity takes the following form,


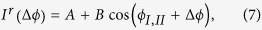


and the contrast of modulation is given approximately as 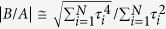. Here *ϕ*_*I,II*_ is phase determined by the choice of the parts *I* and *II*. Setting Δ*ϕ* as −*ϕ*_*I,II*_ makes the total intensity maximum. Since the contribution of each eigenchannel is weighted by the square of *τ*_*i*_, maximization of total intensity preferentially matches the phases of eigenchannels with large *τ*_*i*_. After making the choice of Δ*ϕ* = −*ϕ*_*I,II*_, one can divide the wave into two different segments from the previous ones, and repeat the same process. The sequential application of this maximization process purifies the eigenchannels with high reflectance from an arbitrary incident wave. As a result, the total reflectance increases with the successive iteration of the maximization process.

For the intuitive understanding of the purification process, we describe Eq. (6) using phasor diagrams of various eigenchannels (Fig. 1(b)). We labeled the waves coming from part *I* as phasors in solid blue and those from part *II* in solid red. Note that the length of a phasor, |*c*_*i*_*τ*_*i*_|, tends to be longer for those eigenchannels with higher reflectance (i.e. smaller index *i*). Since eigenchannels are orthogonal, interference occurs only between phasors with the same eigenchannel index. Upon increasing Δ*ϕ*, all the phasors in solid red are rotated by the same amount and become dashed red arrows. In this picture, it is clear that the interference intensity of each eigenchannel is sinusoidally modulated with a period of 2π, and its amplitude of modulation is proportional to *τ*_*i*_^2^. Therefore, the eigenchannels with small indices preferentially contribute to the total interference. As a result, the choice of Δ*ϕ* that maximizes the total intensity preferentially aligns the phasors of eigenchannels with high reflectance.

In order to theoretically validate that the iterative maximization process enriches high-reflection eigenchannels, we simulated the purifying process using a numerically generated reflection matrix based on RMT, which assumes an ideal waveguide geometry^10^. The generated reflection matrix has 300 input channels and the same number of output channels, and the disordered medium is assumed to have about 40% mean reflectance. The Fig. 1(c) shows total reflection as a function of the number of iterations, which increases and then saturates later on. Interestingly, the contrast of modulation, |*B*/*A*|, at each step of iteration is observed to increase (Fig. 1(d)). The increase in the contrast implies that the small number of eigenchannels dominantly contribute to the interference with the increase of total reflectance. For the incident wave obtained after the purification process, we decompose it into reflection eigenchannels and plotted the absolute square of the coefficient for each reflection eigenchannel (Fig. 1(e)). This shows that the contribution of high-reflection eigenchannels increases as a result of purification process. As a comparison, an arbitrary incident wave chosen at the beginning of the iteration has the same contribution by all the eigenchannels.

The schematic diagram of our experiment is shown in Fig. 2. We used a He-Ne laser with the wavelength of 633 nm as a light source and guided the output beam from the laser to a spatial light modulator (the output beam from the laser illuminated a spatial light modulator (SLM, X10468–06, Hamamatsu)) in order to shape the pattern of the beam. The photodetectors, PD1 and PD2, were used to measure the total intensity of the reflected wave from a scattering medium and the intensity of incident wave, respectively. In accordance with the principles described above, we conducted an experiment of preferential coupling waves into reflection eigenchannels with high reflectance. In order to choose parts *I* and *II*, we randomly divided the SLM pixels into two parts as shown in Fig. 3(a). This way of dividing total input channels into two parts was introduced previously^20^ and similar approaches of using multiple input channels in parallel were proposed^21^,^22^, but all these previous methods were applied to optimize intensity at a single output point. Here, half of the pixels, colored blue, constituted part *I* and the other half, colored red, constituted part *II*. Initially, the phase value at each pixel was randomly chosen. The illumination beam was circular with a diameter of 11.4 μm, and the numerical aperture covered by the illumination was 0.91. Therefore, the effective number of orthogonal channels covered in the experiment was about 850. As a disordered medium, we used layers of ZnO particles with 24% average reflectance and thickness of 8 ± 2 μm. To make an overall phase shift Δ*ϕ* for part *II*, the value Δ*ϕ* was added to all the red pixels. The measured total intensity with the increase of Δ*ϕ* indeed exhibited sinusoidal modulation (Fig. 3(b)). For the maximization, we chose four steps of Δ*ϕ* at the interval of π/2 and recorded total intensities, 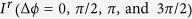. We then obtained coefficients *A*, *B*, and *ϕ*_*I,II*_ of a sinusoidal function, 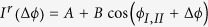, that fits to the four recorded total intensities. We assigned −*ϕ*_*I,II*_ as the first choice of Δ*ϕ* and repeated the process using a new random choice of parts *I* and *II* to further maximize the total reflection. When we applied the iterative feedback control algorithm, the total intensity of reflected wave indeed increased (Fig. 3(c)). Figure 3(d) show the images of reflected wave before and after the feedback control, respectively, which clearly show that the total intensity was dramatically increased. As expected from the theoretical analysis, we observed that the contrast of total intensity modulation with Δ*ϕ* was increased over the iterations (Fig. 3(b)). This is the consequence that the effective number of eigenchannels involved in the interference was reduced. Limiting factors in the amount of reflection increase were found to be the number of SLM pixels and the stability of the experimental setup.

In order to validate that the high-reflection eigenchannels were indeed enriched in the incident wave after the purification process, we measured the reflection matrix of the same medium and obtained all the eigenchannels present in the system. The method is similar to that introduced in our previous study^3^ except that we record reflected wave instead of transmitted wave. We scanned the angle of incident wave at the disordered medium by modifying the slope of phase ramp in the SLM, and recorded complex field map of the reflected wave at each incident angle (Fig. 2). Angular scanning was performed in such a way to uniformly cover the numerical aperture up to 0.91. The number of angles used was 1600. This is about a factor of two oversampling with respect to the number of orthogonal modes at the input, which helped to improve signal to noise ratio of the measurements. The aperture (A) in Fig. 2 was used to match the detection area to illuminated area, and the polarizer (P) to select the same polarization component of scattered wave as the polarization state of the reference beam. The magnification from the disordered medium to the camera was 222. For the recording of a complex field map, a reference beam reflected at BS1 was used to generate an interference pattern at the camera. We used an off-axis digital holography algorithm to extract complex field map out of the interference image. Using 1600 reflected images, we construct a reflection matrix (Fig. 4(a)) and obtained eigenchannels using singular value decomposition (Fig. 4(b)).

Under the same detection configuration for recording the reflection matrix, we performed the maximization of total reflection intensity. We then obtained the contribution of each eigenchannel to the refined incident wave. For this purpose, we calculated the cross-correlation between the refined incident wave and each eigenchannel, and plotted the absolute square of the correlation as a function of the eigenchannel (solid blue in Fig. 4(c)). The contribution of eigenchannels with a smaller index (or higher reflectance) was increased, in good agreement with the theoretical prediction (Fig. 1(e)). The incident wave before the purification process has flat contribution from all the eigenchannels (solid green in Fig. 4(c)). These observations confirm the enrichment of high-reflection eigenchannels.

In contrast to the purification process toward high-reflection eigenchannels, we chose Δ*ϕ* that *minimizes* the total intensity of the reflected waves, i.e. Δ*ϕ* = −*ϕ*_*I,II*_ + π at each iteration step. This minimization process preferentially induced the destructive interference of high-reflection eigenchannels such that low-reflection eigenchannels, i.e. high-transmission eigenchannels, were purified. Detailed numerical analysis is shown in the supplementary information section A. As shown in Fig. 5(a), the reflected wave intensity decreases as the number of iterations was increased. As a disordered medium, we used layers of TiO_2_ particles with 10.9% average transmittance and thickness of 8 ± 2 μm. While minimizing the reflected wave intensity, we simultaneously measured the intensity of the transmitted wave and observed that the transmission is indeed increased (Fig. 5b–e). This validates that we can enrich high-transmission eigenchannels by the control of reflected waves. This is the first *in situ* demonstration of enhancing light energy delivery through a scattering medium. As a reference, we can compare our method with the minimization of the intensity at a single point of the reflected wave. While single-point optimization can enhance transmission to a certain degree, single-point minimization makes negligible contribution to reducing reflectance (Supplementary Fig. S1). Therefore, in the reflection mode, the proposed method is the only possible feedback control approach developed to date. The enhancement of total transmittance for this particular sample was about 2.0, and the maximum enhancement we achieved so far was about a factor of 2.1 (Supplementary Fig. S4).

In our study, there were two inevitable issues that practically limit the achievable enhancement factor. Firstly, we could cover only a fraction of incidence channels at our wavefront shaping process mainly due to the insufficient number of SLM pixels. In terms of numerical aperture, we covered 0.9 out of 1.4, and only a single polarization component was controlled. In total, about 20% of total incidence channels were covered. The limited channel coverage modifies the singular value distribution of the transmission and reflection matrices^3^,^12^,^23^,^24^, which affect to the efficiency of the feedback control. We performed additional simulations accounting for this limited channel coverage (Supplementary Information section C) and observed that the enhancement factor is reduced from 8 to 3.5. Considering that the experimentally observed enhancement factor was about 2.1, significant fraction of discrepancy between the experiment and ideal waveguide geometry can be attributed to this limited channel coverage.

The second issue was the loss of waves scattered away from the view field. In our study, we minimized total reflectance for enhancing total transmission. In this process, some of the reduced reflectance can be converted to loss of waves scattered away from the view field. Considering that the addition of transmittance and reflectance was about 0.55, not unity, there was approximately 45% of loss in our experiment. Since it is extremely difficult to model the effect of this scattering loss at present, it is difficult to quantify its effect to the feedback control. If we assume this effect as a loss in the detection channel, we expect the reduction of enhancement factor from 3.5 to 2.8. The residual discrepancy might come from the system perturbation and phase control error of the SLM.

The proposed method of iterative operation can in principle be much faster than the previous method of recording a matrix. In our study, the maximization process took about 30 minutes for 1000 iterations because we used a liquid-crystal based SLM whose effective frame rate is only 10 Hz. The use of a faster wavefront shaping device such as a digital micromirror device (DMD) will greatly reduce the operation time^25^,^26^.

To conclude, we have demonstrated that the coupling of incident wave to reflection eigenchannels can be controlled in such a way that the eigenchannels of either high or low reflection can be preferentially excited. This has led to the control of wave transmission by the use of reflected wave. Sensing, imaging, and manipulating a target hidden under highly scattering layers are universal problems where efficient illumination to the target is prerequisite condition. Our study of exploiting reflection eigenchannels will facilitate efficient light energy delivery in the realistic environments where sample perturbations are present, and our method will readily find numerous important applications. For example, the proposed method can increase the working depth of optical imaging such as diffuse optical tomography^27^ and photoacoustic tomography, and increase the treatment depth of phototherapies^28^,^29^ and laser surgeries, and facilitate deep-tissue optical manipulations such as in optogenetics^30^,^31^. Since the methodology works for all types of waves, its applicability can be extended to microwave^32^ and sound wave technologies^33^.

## Additional Information

**How to cite this article**: Choi, W. *et al.* Preferential coupling of an incident wave to reflection eigenchannels of disordered media. *Sci. Rep.* **5**, 11393; doi: 10.1038/srep11393 (2015).

## Supplementary Material

Supplementary Information

## Figures and Tables

**Figure 1:**
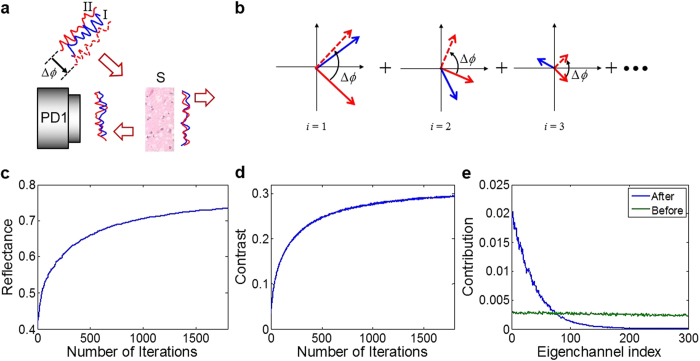
Theoretical analysis of purifying reflection eigenchannels with high reflectance. **a**, Schematic diagram of maximization process. The incident wave was decomposed into two parts indicated by *I* and *II*, and the second part is shifted in phase by Δ*ϕ*. A detector sensor (PD1) records the reflected wave by a scattering sample (S). **b**, Phasor diagrams for the reflection eigenchannels. From left to right, the eigenchannel index is increasing such that the length of phasor decreases. Red and blue phasors indicate waves coming from parts *I* and *II* of the incident wave, respectively. Dashed red arrows show the phasors of solid red arrows after a phase shift of Δ*ϕ* **c**, Total reflectance with the increase of the number of iterations. **d**, Contrast of modulation with respect to Δ*ϕ* at each step of iteration. e, Square of normalized correlation between an incident wave and eigenchannels at the input plane. Green and blue curves correspond to the incident waves before and after the purification process, respectively.

**Figure 2:**
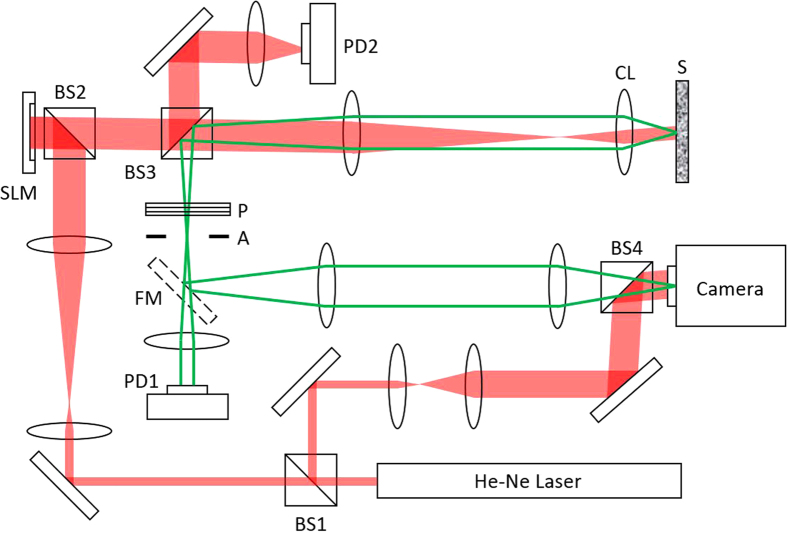
Schematic diagram of experimental setup. He-Ne Laser (Melles Griot 25-LHP-928–230), BS1-4: Beamsplitters, SLM: Spatial Light Modulator (Hamamatsu Photonics, X10468–06), PD1 and PD2: photodiodes (THORLABS, PDA36A–EC), S: disordered medium, CL: condenser lens (Nikon, Achr-Apl N.A = 1.4), FM: flip mirror, Camera (RedLake M3), P: polarizer, and A: aperture. Reflected wave from the disordered medium to the detector sensors was indicated as green lines for clarity although its wavelength is the same as that of incident wave.

**Figure 3:**
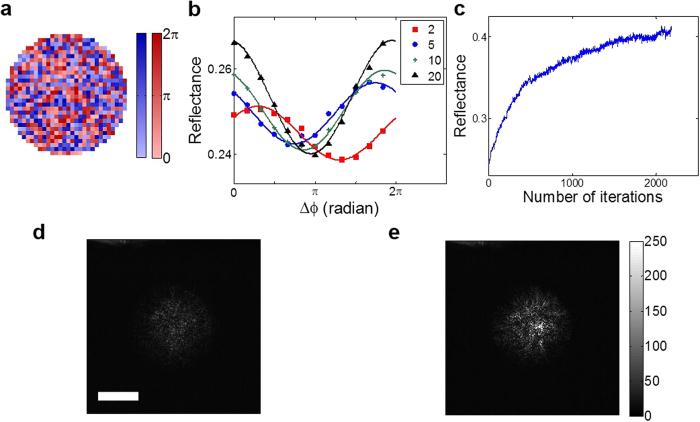
Experimental results of the preferential coupling to high-reflection eigendmoes. **a**, Representative phase pattern written on SLM. Pixels colored in blue constitute part *I*, and those in red constitute part *II*. Color bar, phase in radians. **b**, Experimentally measured total reflection as a function of Δ*ϕ* at the iteration steps of 2, 5, 10, and 20. **c**, Experimentally measured reflectance over the increase of the number of iterations. **d** and **e**, The intensity images of the transmitted wave before and after the purification process, respectively. Scale bar, 5 μm. Color bar indicating intensity in arbitrary units applies to both **d** and **e**.

**Figure 4:**
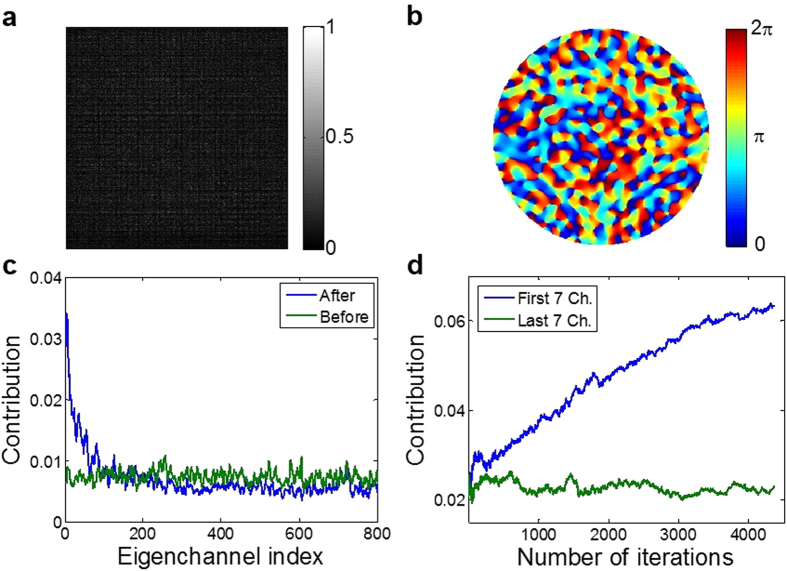
Enrichment of high-reflection eigenchannels due to the purification process. **a**, Amplitude part of the reflection matrix recorded by the setup shown in Fig. 2. Column and row indices indicate input and output channels, respectively. **b**, One of the representative reflection eigenchannels having the highest reflectance. Only the phase map is shown here, and there is an associated amplitude map. **c**, The contribution of each eigenchannel to the incident wave before (green) and after (blue) the purification process. **d**, The contribution to the incident wave of the first 7 eigenchannels (blue) and last 7 eigenchannels (green) with respect to the number of iterations.

**Figure 5:**
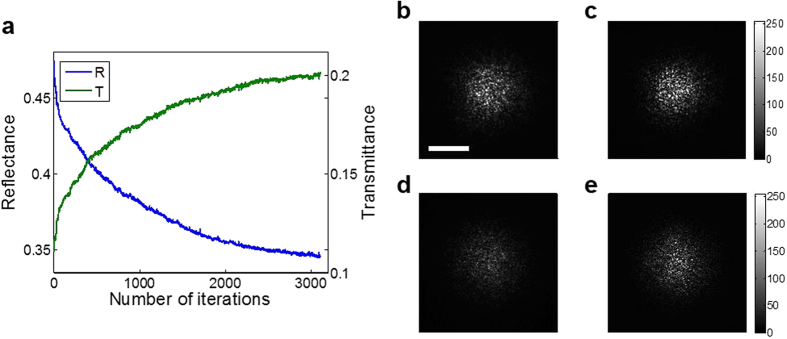
Transmission enhancement by the purification of reflection eigenchannels with low reflectance. **a**, Total reflectance with the increase of the number of iterations (blue) and total transmittance (green) measured at the same time. **b** and **c**, Intensity images of the reflected wave before and after the purification process. **d** and **e**, Intensity images of the transmitted wave before and after the purification process. Scale bar, 10 μm. Color bar indicates intensity in arbitrary units.
