# Supplementary material for: Preferential coupling of an incident wave to reflection eigenchannels of disordered media
Source: Sci Rep. 2015 Jun 16;5:11393. doi: 10.1038/srep11393 (PMC4650648; doi:10.1038/srep11393)
Supplement: Supplementary Information [file srep11393-s1.pdf]

Preferential coupling of an incident wave to reflection eigenchannels of disordered media

Wonjun Choi[1], Moonseok Kim[1], Donggyu Kim[1], Changhyeong Yoon[1], Christopher Fang-Yen[1,2], Q-Han Park[1] and Wonshik Choi[1,3*]

[1]*Department of Physics, Korea University, Seoul 136-701, Korea*

[2]*Department of Bioengineering, University of Pennsylvania, Philadelphia, Pennsylvania 19104, USA*

[3]*Center for Molecular Spectroscopy and Dynamics, Seoul 136-701, Korea*

A. Numerical simulation of enriching low-reflection eigenchannels and its comparison with single-channel minimization.

In order to theoretically validate that purification of low-reflection eigenchannels enhances transmitted wave intensity, we simulated the purifying of low-reflection eigenchannels by using both reflection and transmission matrices generated by the RMT, which are coupled together. As can be seen in Fig. S1(a), the reflected wave intensity decreases with the increase of the number of iterations. In accordance with the decrease in reflected wave intensity, transmitted wave intensity is increased. Similar to the case of maximizing reflected intensity, the contrast of modulation of reflected intensity is increased as the number of iterations is increased (Fig. S1(b)). This implies that eigenchannels are refined and thus smaller number of eigenchannels are getting involved in the interference. As a result, the contribution of low-reflectance eigenchannels has increased after the minimization (Fig. S1(c)). Interestingly, the single-point minimization alters the distribution very little, and thus makes almost no effect on reducing the reflection. As a consequence, the enhancement of transmission is negligible.

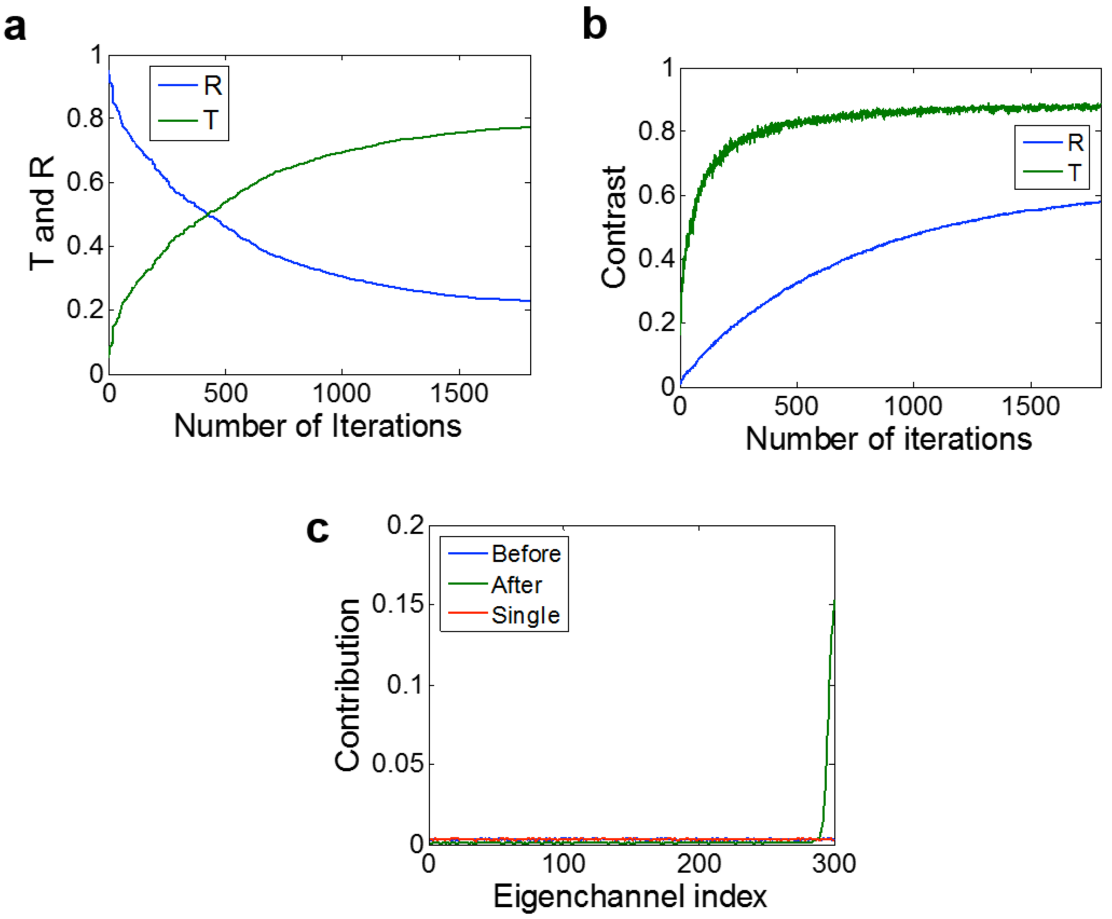

Figure S1. Effects of iterative feedback control on the total intensity of reflective wave, contrast of modulation, and the contribution of reflection eigenchannels to the incident wave. (a) Reflected wave intensity with the increase of the number of iterations. After each iteration, the resulting incident wave is multiplied by the transmission matrix to obtain transmitted wave intensity. Due to the energy conservation, the transmittance is increased by the minimization of reflectance. (b) The contrast of modulation of reflected intensity (green) at each step of iteration. In conjunction with the increased contrast of modulation of reflected intensity, the contrast of modulation of transmitted intensity is also increased (blue). (c) The absolute square of the normalized correlation between the incident wave and reflection eigenchannels at the input plane. Blue, green, and red curves correspond to the incident waves before the minimization, after the minimization, and after the single-channel minimization, respectively.

B. Increase of the contrast of modulation

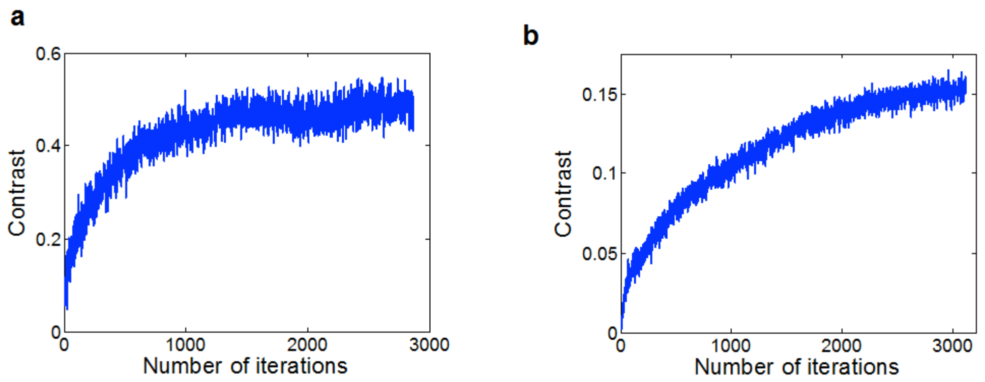

Figure S2. Experimentally measured contrast of modulation. (a) The contrast of modulation of transmitted intensity at each step of iteration in the transmission mode of experiment. (b) The contrast of modulation of reflected intensity at each step of iteration in the reflection mode of experiment.

Numerical analysis described in section A shows that the contrast of modulation of reflected intensity is expected to increase with the progress of iteration. We observed this tendency in our experiment as well. In Fig. 3(b) in the main text, we presented the modulation of reflected intensity at a few representative numbers of iterations. Here in Fig. S2, we provided the change in the contrast of modulation of reflected and transmitted intensity for all the iterations steps.

C. Effect of limited channel coverage on the purification of low-reflection eigenchannels

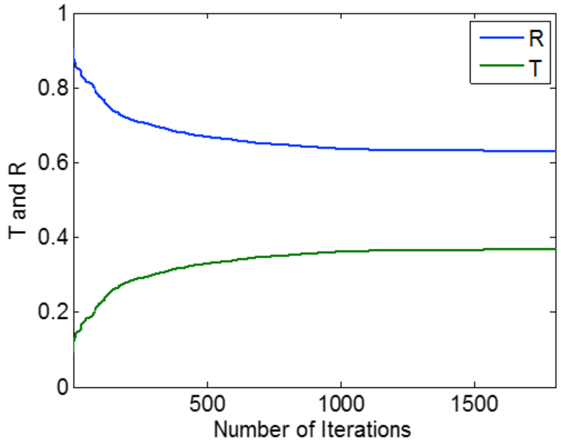

Figure S3. Effect of limited channel coverage on the performance of the iterative feedback control for the total intensity of reflective wave. Blue curve shows reflected wave intensity with the increase of the number of iterations. After each iteration, the resulting incident wave is multiplied by the transmission matrix to obtain the intensity of transmitted wave (green curve). Due to the limited channel coverage, the feedback process is less effective than the ideal case shown in Fig. S1. The enhancement of total transmittance was about 3.5.

In our feedback control experiment, the SLM covered numerical aperture of 0.9 out of 1.4 and only single-polarization component was controlled. Therefore, about 20 % of total input channels were covered. On the detection side, we collected reflected wave intensity by the full numerical aperture of the condenser lens and all the polarization components were collected. In order to investigate the effect of limited channel coverage, we constructed a numerical reflection and transmission matrices and cropped out the 80 % of input channels. For these truncated matrices, we performed numerical feedback control, and obtained reflectance and transmittance at each iteration (Fig. S3). We could observe that the limited channel coverage significantly undermines the effectiveness of the feedback control. This is mainly because the singular value distribution is modified depending on the channel coverage. As the channel coverage is reduced, the distribution departs from sech function and becomes a monotonic and linear decay curve. Therefore, The difference of singular values between low-reflection and high-reflection channels is reduced.

D. The best transmission enhancement observed in the reflection mode of experiment

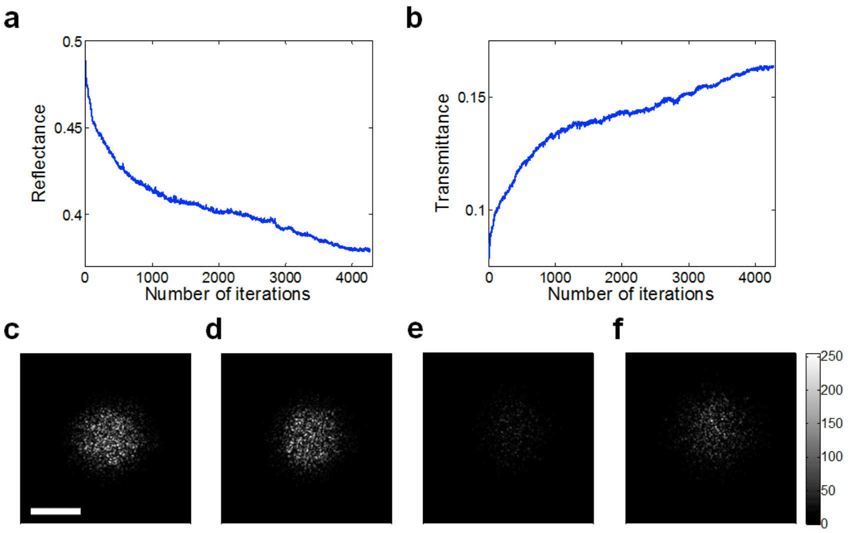

Figure S4. Transmission enhancement for a sample presenting the best experimental record. (a) Total reflectance depending on the number of iterations. (b) Total transmittance depending on the number of iterations. (c) and (d) Intensity images of the reflected wave before and after the feedback control. (e) and (f) Intensity image of the transmitted wave before and after the feedback. Scale bar, 10 μm. Color bar indicates intensity in an arbitrary unit.

We performed the reflection mode of experiment for the disordered media with various average transmittances. The figure S4 show the experimental data for a disordered medium with average transmittance of 7.9 %, which shows the best experimental record of enhancement factor, 2.1.

E. Comparison between the total-intensity optimization and single-point optimization in terms of total transmittance

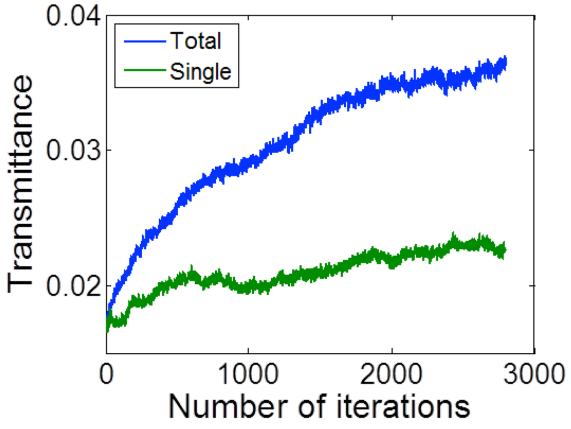

Figure S5. Comparison between the feedback control of total intensity and optimization of intensity at a single point. Transmittance with the increase of the number of iterations for total-intensity feedback control (blue) and single-point optimization (green).

Our theoretical analysis predicts that the proposed method is superior to the previously reported single-point optimization method. In Fig. S5, we show the comparison between the two methods. While the single-point optimization method increased the total transmittance by 28.6 % compare to average transmittance, our proposed method enhanced the total transmittance by 118 %. This new method offered four times more enhancement than the single-point optimization at this particular sample.
